# A homage to Professor Adolpho Hoirisch (1930-2023), a luminary of Brazilian psychiatry, and to his humanitarian and public commitment

**DOI:** 10.47626/2237-6089-2023-0769

**Published:** 2024-08-14

**Authors:** Antonio E. Nardi, Mauro V. Mendlowicz

**Affiliations:** 1 Universidade Federal do Rio de Janeiro Rio de Janeiro RJ Brazil Universidade Federal do Rio de Janeiro, Rio de Janeiro, RJ, Brazil.; 2 Universidade Federal Fluminense Niterói RJ Brazil Universidade Federal Fluminense, Niterói, RJ, Brazil.

Professor Adolpho Hoirisch, a distinguished figure in all fields of psychiatry, passed away recently, leaving behind a remarkable legacy that spans a lifetime of contributions to these disciplines. He was born on May 22, 1930, in Bento Ribeiro, a tranquil neighborhood in the North Zone of Rio de Janeiro, Brazil. His parents, Nathan and Perlea Hoirisch, were Jewish immigrants from Bessarabia, a region marked by a turbulent history. Nestled between powerful and chronically warring neighbors, such as Russia and Romania, the population of Bessarabia found themselves caught in the crossfire. They were often forbidden from speaking Romanian during Russian occupation and Russian during Romanian control. The history and geography of Bessarabia played a pivotal role in shaping young Adolpho’s mindset and activities.

The tumultuous history of his parents’ homeland instilled in him a deep appreciation for justice, freedom, and the fundamental rights of individuals. This profound sense of justice was further influenced by the injustices he witnessed during the Estado Novo era dictatorship in Brazil, when the political police brutally invaded his family’s home, searching for evidence of his father’s alleged communist ties, and left an enduring mark on Adolpho Hoirisch. These experiences fueled his unwavering commitment to the principles of human rights and social justice, which became integral to his professional life.

Adolpho Hoirisch studied at the nearby Colégio Souza Marques and graduated from high school in 1948. Although his family had been involved in commerce and banking in Europe, he chose to become a physician and managed to enroll at the prestigious Brazilian National School of Medicine (Faculdade Nacional de Medicina). While at medical school, he trained under the supervision of noted psychiatrists such as professors Mauricio de Medeiros and José Leme Lopes. He graduated from medical school in 1954. Already fully enthralled by psychiatry, in 1955, against the advice of his mother (who wanted him to pursue a career as a laboratory physician), he decided to become a trainee on the renowned psychiatry training program sponsored by the National Division of Mental Health (DINSAM - Divisão Nacional de Saúde Mental) of the Brazilian Ministry of Health at the Centro Psiquiátrico D. Pedro II clinic. After completing the 1-year training, Adolpho Hoirisch was invited to join the psychiatry training program, this time as an assistant professor.

Adolpho Hoirisch started to build a career in the public sector in 1955 when he joined DINSAM as a psychiatrist stationed at the Heitor Carrilho Judicial Asylum (Manicômio Judiciário Heitor Carrilho). There, he delved into the minds of mentally deranged criminal offenders and conducted assessments of criminal responsibility and dangerousness. From 1958 to 1962, he served in the Brazilian Air Force as a medical officer specializing in neuro-psychiatry.

In 1965, Adolpho Hoirisch took his first steps towards pursuit of an academic career in psychiatry. He contacted professor Leme Lopes, the head of the Psychiatry Institute (Instituto de Psiquiatria) at the Universidade Federal do Rio de Janeiro (UFRJ), and volunteered to work as an unpaid clinical instructor. The next year, however, a new federal law unexpectedly promoted all clinical instructors to assistant professors and thus consolidated Adolpho Hirsch’s lifelong commitment to the UFRJ.

In 1970, Adolpho Hoirisch completed his psychoanalytic training and became an associate member of the Rio de Janeiro Psychoanalysis Society (SPRJ - Sociedade Psicanalítica do Rio de Janeiro). Four years later, he achieved the prestigious status of SPRJ full member and teaching analyst.

Also in 1970, Adolpho Hoirisch was awarded a Senior Lecturer position ( *livre-docência* ) in psychiatry by the UFRJ. To achieve this top university post, the candidate must demonstrate that she or he fulfills the university’s set criteria of excellence in research and teaching. A precondition is publication of numerous research articles during the preceding years. Additionally, the qualification process includes a written examination, the candidate must deliver a public lecture and submit a doctoral thesis, and the process culminates in a *viva voce* examination.

Adolpho Hoirisch’s next achievement was his appointment as the first-ever Full Professor of medical psychology at the UFRJ, after a very competitive public selection process against some of the most brilliant minds in the field in the city of Rio de Janeiro. His thesis for the full professor selection process, “The Question of Medical Identity,” dealt with the challenges that physicians face when relinquishing their professional roles when they get sick. This was an ahead-of-its-time revolutionary subject that only decades later would be given due acknowledgment.

The newly created medical psychology chair required a new class of psychiatrists and specialists in mental health and it was up to Professor Adolpho Hoirisch to select, train, and supervise them. He created and directed a pioneering medical psychology service at the UFRJ’s Hospital Universitário Clementino Fraga Filho, providing psychiatric and psychological assessment and treatment for inpatients and outpatients and also for the medical staff. Professor Adolpho Hoirisch also paid particular attention to the psychological needs of the medical students. He instituted a Psychopedagogy and Professional Guidance Program (POPPE - Programa de Orientação Psicopedagógica e Profissional) at the UFRJ School of Medicine, aiming to help troubled medical students overcome evolving crises and doubts about personal, vocational, and sexual identity and, thus, recover their pleasure in learning, as well as correct deficits in their learning process.^1^ Again, this was another ahead-of-its-time subject that only more recently was given due acknowledgment.^2^

**Figure 1:**
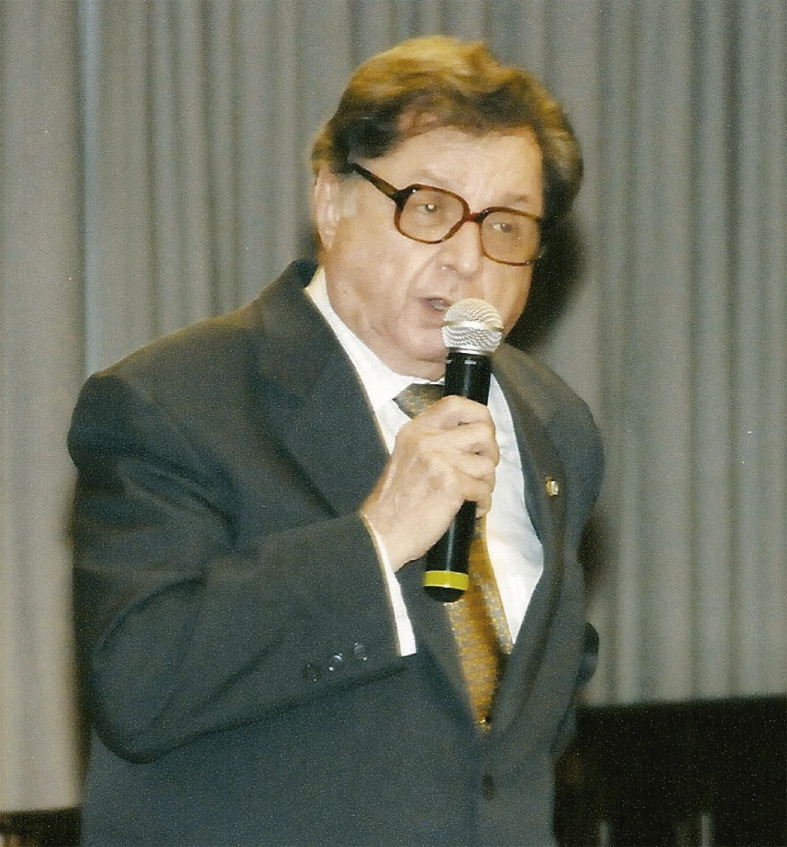
Emeritus Professor Adolpho Hoirisch during one of his lectures at the National Academy of Medicine, Brazil, in 2011.

In 1988, Professor Adolpho Hoirisch was elected a full member of Brazil’s National Academy of Medicine (Academia Nacional de Medicina) ( Figure 1 ), thus cementing his place among the most distinguished physicians in Brazil. As a prerequisite for acceptance into the National Academy of Medicine, he submitted a memoir entitled “The Psychiatric Implications of Iatrogenesis.” He occupied chair # 46, whose patron is Afrânio Peixoto.

Professor Adolpho Hoirisch was also affiliated to numerous national and international scientific societies, such as the Rio de Janeiro State Psychiatry Association (Associação Psiquiátrica do Estado do Rio de Janeiro), the Brazilian Psychiatric Association, the World Psychiatric Association, the Rio de Janeiro Psychoanalysis Society, and the International Psychoanalytic Association (IPA), among others. He was also a full member of the Brazilian Academy of Military Medicine (Academia Brasileira de Medicina Militar).

During his academic and professional career, Professor Hoirisch conducted a series of important studies making significant contributions to the fields of psychiatry and medicine. Some of his most notable works include “Freud y las vicisitudes de una sociedad psicoanalítica,”^3^ “From the viewpoint of a psychiatric community: The hospital,”^4^ “L’unité psychiatrique comme corps étranger dans l’hôpital général,”^5^ “Psiquiatria e violência,”^6^ “Termos médicos e mitologia greco-romana,”^7^ “O paradigma psicossomático: visão de um psiquiatra,”^8^ and “A relação médico-paciente e o impacto tecnológico.”^9^ These studies demonstrated his dedication to advancing our understanding of mental health and the intricate relationship between psychological and medical disciplines. In his work, Adolpho Hoirisch exhibited a rare combination of academic rigor and genuine compassion for the individuals under his care.

Even after retiring from the UFRJ, Adolpho Hoirisch continued to contribute to the field of psychiatry, joining the faculty of the School of Medicine at the Universidade Gama Filho, where he and his assistants spent a decade lecturing on psychiatry, mental health, and medical psychology. His passion for education remained undiminished, and he generously shared his knowledge and expertise with the next generation of medical professionals. He was also a very active member of the special committee of the Regional Board of Medicine of the State of Rio de Janeiro (CREMERJ - Conselho Regional de Medicina do Estado do Rio de Janeiro) whose main goal was to address the mental health of doctors. In 2013, he received a much-deserved honor from UFRJ, which awarded him the title of Professor Emeritus. This was an acknowledgment of his lifelong commitment to the fields of psychiatry, psychoanalysis, and medical education, his devotion to the psychological well-being of individuals ranging from criminal offenders to medical students, and his exploration of the intricacies of the issue of identity – fostered by his family’s history and his own experiences – all of which have greatly enriched our understanding of the integrality of the human being.

Professor Adolpho Hoirisch was a remarkable individual whose life was dedicated to the pursuit of knowledge, the betterment of society, and the advancement of the medical and psychiatric fields. He was a passionate advocate for justice, human rights, and the well-being of individuals. His contributions, as both practitioner and educator, have left an indelible mark, and his legacy will continue to inspire future generations in the realms of psychiatry and medicine. He was also a very warm individual, an inspiring teacher, a charming conversationalist, an exceptional joke teller, and a true friend to his friends. We, his former disciples and, later, his junior colleagues, will miss him dearly.
